# Spinal Mechanisms of Testosterone Involved in Itch Regulation

**DOI:** 10.1002/cns.71196

**Published:** 2026-10-02

**Authors:** Luying Chen, Xiaodong Wang, Lu Cheng, Pengtao Li, Zhenyu Wu, Hui Li

**Affiliations:** ^1^ Department of Human Anatomy, Histology and Embryology and K.K. Leung Brain Research Center Air Force Medical University Xi'an ShaanXi China; ^2^ Department of Emergency Medicine Inner Mongolia Autonomous Region Corps Hospital of PAP Hohhot Inner Mongolia China

**Keywords:** androgen receptor, itch, sex difference, spinal dorsal horn, testosterone

## Abstract

**Background and Objective:**

Clinical and epidemiological data demonstrate that females exhibit higher itch sensitivity than males, and prior research has confirmed estrogen exacerbates pruritus to drive this sexual dimorphism. Nevertheless, it remains poorly defined whether testosterone, the primary androgen, exerts anti‐pruritic effects and contributes to sex disparities in itch perception. This study therefore has three primary objectives: (1) to characterize testosterone's regulatory effects on acute and chronic itch across male and female mice; (2) to identify spinal androgen receptor‐expressing neuronal populations mediating testosterone's anti‐pruritic actions; (3) to elucidate the downstream GABAergic synaptic cascade by which testosterone suppresses spinal itch transmission.

**Methods:**

C57BL/6 mice and GRPR‐GFP mice were used in this study. Behavioral tests were performed to evaluate the effects of exogenous testosterone administration, orchiectomy, androgen receptor (AR) antagonist flutamide, and GABA_A_ receptor (GABA_A_R) antagonist bicuculline on acute itch induced by histamine or chloroquine and chronic itch induced by 2,4‐dinitrofluorobenzene (DNFB), as well as anxiety‐like behaviors assessed by elevated plus‐maze (EPM) and open‐field test (OFT). Double immunofluorescence staining was applied to detect the co‐localization of AR and PAX2 in the spinal dorsal horn. Whole‐cell patch‐clamp electrophysiology was used to record synaptic transmission and excitability of GRPR^+^ neurons in spinal cord slices.

**Results:**

Testosterone contributed to sex differences in itch in mice. Exogenous testosterone suppressed histamine‐ or chloroquine‐evoked acute itch and alleviated DNFB‐induced chronic itch and anxiety‐like behaviors. Orchiectomy enhanced itch sensitivity, which was reversed by testosterone replacement. AR was predominantly expressed on PAX2^+^ GABAergic inhibitory interneurons in the superficial spinal dorsal horn. Testosterone increased spontaneous inhibitory synaptic input and reduced excitability of GRPR^+^ neurons. The antipruritic effect of testosterone was blocked by intrathecal flutamide or bicuculline.

**Conclusion:**

Testosterone inhibits itch transmission at the spinal level by activating AR on GABAergic interneurons, enhancing GABA release, and subsequently suppressing GRPR^+^ neurons via GABA_A_R. Testosterone acts as an endogenous antipruritic hormone and contributes to the sex difference in itch perception, providing a novel target for clinical itch treatment.

## Graphical abstract

Testosterone serves as an endogenous anti‐pruritic hormone underlying sex‐dependent itch differences. In the spinal dorsal horn, testosterone binds androgen receptors on GABAergic interneurons to enhance GABA release, suppressing GRPR^+^ − pruriceptive neurons through GABA_A_ receptors and alleviating acute/chronic itch alongside itch‐comorbid anxiety. This spinal circuit offers a novel target for pruritus therapy.

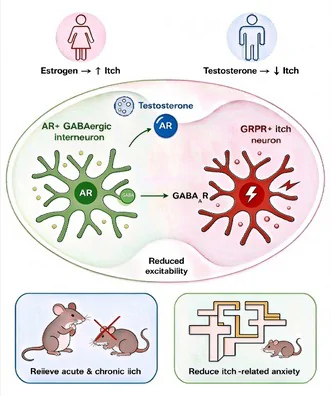

## 1 Introduction

Itch, the sensation that elicits the urge to scratch, is a common experience in daily life. It ranges from acute itch caused by transient skin irritation to chronic, persistent itch, which may present as localized, well‐defined epidermal discomfort or diffuse perceptual abnormalities. As a defensive alarm system conserved through evolution, acute itch serves a protective function by prompting scratching to avoid potential irritants or signal underlying issues. In contrast, chronic itch is often a symptom of systemic disease. It frequently leads to sleep disturbances and emotional disorders, thereby initiating a vicious cycle wherein pruritus and negative emotions exacerbate each other.

Clinical epidemiological data demonstrate that pruritic sensitivity, alongside the prevalence of anxiety and depressive disorders, is generally higher in women than in men [1, 2]. This disparity suggests a potential role for sex hormones. Previous studies have demonstrated that estrogen promotes itch and is a key factor underlying the significantly higher itch sensitivity observed in female mice compared to males [3, 4]. This raises important questions: Could testosterone, as the primary androgen, also contribute to sex differences in pruritus? Does it participate in the regulation of itch sensation? Currently, systematic experimental investigation into this possibility is lacking.

The spinal dorsal horn (SDH) is the primary relay center for transmitting pruriceptive signals from the periphery to the brain. Local neural circuits within the SDH are crucial for modulating these signals. The established spinal itch transmission pathway involves peripheral stimuli activating natriuretic peptide B (NPPB)‐positive neurons in the dorsal root ganglia. These neurons release NPPB to activate spinal gastrin‐releasing peptide (GRP)‐ergic neurons expressing the NPRA receptor. The GRP‐ergic neurons then release GRP to excite gastrin‐releasing peptide receptor (GRPR)‐positive neurons in the SDH, thereby conveying the itch signal from the periphery to the spinal cord [1]. GRPR‐positive neurons indirectly transmit itch signals to the parabrachial nucleus via spinal projection neurons, and subsequent relay to the thalamus and other brain regions underlies the conscious perception of itch [5]. As the first identified itch‐specific receptor, GRPR‐positive neurons represent a critical hub in the spinal itch circuit. Genetic deletion of the *Grpr* gene or selective ablation of these neurons markedly reduces scratching behaviors in mice, making it a central focus for studies on itch transmission and modulation mechanisms [6, 7].

The clinical relevance of itch‐related sex differences is underscored by epidemiology. The prevalence of pruritic skin diseases is generally higher in women. Surveys indicate that approximately 27% of adults experience pruritus, with rates significantly higher in females than in males [8, 9]. Chronic pruritus severely impairs quality of life and is closely linked to anxiety and depression, creating a detrimental cycle [10]. A specific survey of psoriasis patients (*n* = 1979) found that 61.3% experienced disease‐related itch, with female patients reporting significantly greater burden from chronic pruritus symptoms than males [11].

Mechanistically, estrogen binding to ERα enhances itch perception and is the primary driver of higher pruritus sensitivity in females [3, 12]. Another study found that estradiol upregulates spinal expression of the itch mediator GRP and increases the responsiveness of GRPR‐positive neurons to histamine. The pruritus‐promoting effect of estradiol was abolished by intrathecal injection of a GRPR antagonist [3]. Thus, estrogen selectively potentiates itch perception in female mice via the spinal GRP/GRPR system. These findings collectively indicate that females have a lower threshold for itch perception and face greater therapeutic challenges. However, it remains unknown whether the sex difference in itch is also associated with a potential anti‐pruritic effect of androgens like testosterone, beyond the established pro‐pruritic role of estrogen.

Testosterone (T), a steroid hormone secreted mainly by the testes in males and the ovaries in females (with minor adrenal production), is a neuroactive steroid. It regulates various neurotransmitter systems, including serotonin (5‐HT), dopamine, and gamma‐aminobutyric acid (GABA), which may underlie its functional mechanisms [13, 14]. Notable sex differences exist in conditions like anxiety, depression, and pain, with females experiencing roughly twice the prevalence of males. Sex hormones are considered a key factor in this disparity [15]. Given that testosterone has demonstrated analgesic, anxiolytic, and antidepressant properties [13, 16, 17, 18], it is plausible that testosterone, alongside estrogen, contributes to the observed sex differences in itch.

In light of the above reasons, the present study will employ morphological, behavioral‐pharmacological, and electrophysiological techniques to investigate the effects of testosterone on itch perception at both peripheral and spinal levels. We aim to elucidate the underlying receptor mechanisms and spinal circuit involvement, and to further explore whether testosterone can ameliorate the comorbid state of itch and anxiety. Collectively, these findings are expected to provide novel insights for the clinical management of pruritus and the development of targeted therapeutics.

## 2 Materials and Methods

### 2.1 Animals

C57BL/6 mice (8–12 weeks) were obtained from the Laboratory Animal Management Center of Air Force Medical University. GRPR‐GFP mice were kindly provided by Prof. Zhou‐Feng Chen (Washington University in St. Louis, USA). All animals were housed in a specific‐pathogen‐free (SPF) barrier facility under a strict 12‐h light/dark cycle. Temperature and humidity were maintained within optimal ranges to minimize environmental stress. Mice were provided with ad libitum access to standard chow and sterilized water, with a maximum of six animals per cage. All animal experimental procedures complied fully with the institutional ethical guidelines for the care and use of laboratory animals.

### 2.2 Effect of Testosterone on Itch Behavior in Normal Mice

Normal male and female mice were given daily abdominal subcutaneous injections of 20 μL testosterone solution (HY‐B1269, MedChemExpress, USA) at concentrations of 25, 50, and 75 μg/μL over three consecutive days to form the testosterone‐treated (T) experimental group. Control mice received 20 μL of the vehicle, pure olive oil. Following this pretreatment, acute itch was induced by intradermal injection of histamine or chloroquine into the nape of the neck. Itch‐related scratching behavior was recorded for 30 min using a video monitoring system. A scratching bout was defined as hind limb elevation directed toward the injection site, followed by limb return to the cage floor or licking behavior. All videotaped scratching behaviors were quantified by investigators blinded to experimental group allocations.

### 2.3 Effect of Castration on Itch Behavior in Male Mice

#### 2.3.1 Bilateral Orchiectomy Model

Male C57BL/6 mice were anesthetized by inhalation of isoflurane. The scrotal area was shaved and disinfected. With the mouse in a supine position, a 0.5 cm midline incision was made along the scrotal raphe. After blunt dissection, adipose tissue was removed to expose the testes. The upper and lower poles of each testis (connected to the epididymis and the vascular supply/vas deferens, respectively) were ligated with sutures. Both testes were excised, and the remaining tissues were returned to the abdominal cavity. The skin incision was sutured and disinfected. Postoperative mice were kept on a heating pad until recovery of spontaneous activity before being returned to their home cages.

#### 2.3.2 Itch Behavior in Castrated Mice

Male mice were randomly assigned to three groups: a sham‐operated group (ShamV), where the scrotal incision was immediately sutured; an orchiectomy + olive oil group (Orx + olive); and an orchiectomy + testosterone group (Orx + T). After 7 or 20 days of recovery, mice in the ShamV and Orx + olive groups received subcutaneous abdominal injections of olive oil for 3 days, while the Orx + T group received testosterone injections. Acute itch was then induced with intradermal histamine or chloroquine. Scratching bouts over 30 min were recorded and defined as above.

### 2.4 Effect of Testosterone Receptor Antagonism

C57BL/6 mice were randomly divided into four groups. One day prior to the experiment, the nape and back skin were shaved. Under brief inhalation anesthesia, mice received an intrathecal injection (10 μL volume) at the L5‐L6 intervertebral space. Group 1 (Control) received the vehicle (ethanol: saline = 1:14). Group 2 (Flu+T) received a mixed solution of testosterone (5 mmol/L) and the androgen receptor antagonist flutamide (HY‐B0022, MedChemExpress, USA; 10 mmol/L). Group 3 (T) received testosterone alone (5 mmol/L), and Group 4 (Flu) received flutamide alone (10 mmol/L). Thirty minutes postinjection, after confirming full recovery from anesthesia, acute itch was induced by intradermal histamine injection. Scratching bouts over the subsequent 30 min were recorded and analyzed as described.

### 2.5 Effect of Testosterone on Itch and Anxiety‐Like Behaviors in a Chronic Itch Model

#### 2.5.1 DNFB Model Construction and Itch Behavior Assessment

Male or female C57BL/6 mice were randomly selected for establishing a chronic itch model using 2,4‐dinitrofluorobenzene (DNFB). Following successful sensitization and challenge, mice were placed in a behavioral arena for observation. Modeling success was verified by video recording, defined as exhibiting more than 110 (male) or 140 (female) scratching bouts within 30 min. Successfully modeled mice were then divided into two subgroups. Mice in the experimental group (DNFB+T) received consecutive subcutaneous abdominal injections of testosterone solution for 3 days. Control mice (DNFB) received subcutaneous injections of the vehicle (olive oil) for the same duration. Subsequently, behavioral assessment was conducted, which included video recording of spontaneous scratching, followed by the elevated plus‐maze (EPM) test and the open‐field test (OFT) to evaluate anxiety‐like behaviors.

#### 2.5.2 Effect of Intermittent Testosterone Administration During DNFB Modeling

Male C57BL/6 mice were randomly allocated to two groups: a control group (DNFB) receiving intermittent subcutaneous abdominal injections of olive oil, and an experimental group (DNFB+T) receiving intermittent injections of testosterone. Starting from day 3 of the DNFB sensitization protocol, mice received their respective injections once every 2 days throughout the model establishment phase. After the chronic itch model was fully established, comprehensive behavioral assessment was performed sequentially via video recording, the EPM test, and the OFT.

### 2.6 Double Immunofluorescence Staining for AR and PAX2 in the Spinal Dorsal Horn

Adult male C57BL/6 mice were deeply anesthetized via inhalation of isoflurane. The vascular system was first perfused with 0.01 M phosphate‐buffered saline (PBS) for rinsing, followed by fixation with 200 mL of 4% paraformaldehyde. After perfusion, the lumbosacral enlargement of the spinal cord was dissected and post‐fixed in 4% paraformaldehyde for at least 4 h. The tissue was then cryoprotected through sucrose gradient dehydration for a minimum of 48 h. Transverse serial sections (30 μm thick) were cut on a cryostat and collected in 0.01 M PBS. Sections were blocked with 10% normal fetal bovine serum (FBS) to reduce non‐specific binding. They were then incubated with a primary antibody mixture containing mouse anti‐androgen receptor (AR) antibody (YM6817, Immunoway, USA; 1:100) and rabbit anti‐PAX2 antibody (PAX2‐231AP, Life technologies, USA; 1:100). Following washes, sections were incubated with a secondary antibody mixture consisting of biotin‐conjugated donkey anti‐rabbit IgG (AP182B, Merck Millipore, USA; 1:500) and Alexa Fluor 488‐conjugated donkey anti‐mouse IgG (A21202, Invitrogen, USA; 1:500). Finally, sections were incubated with Alexa Fluor 594‐conjugated avidin (S32356, Invitrogen, USA; 1:1000) diluted in 0.01 M PBS. After thorough washing, sections were mounted on glass slides, air‐dried in the dark, and coverslipped with a DAPI‐containing fluorescent mounting medium (1:800). Stained sections were stored in the dark and imaged using a laser scanning confocal microscope (Olympus FV1000).

### 2.7 Whole‐Cell Patch‐Clamp Recording

#### 2.7.1 Preparation of Spinal Cord Slices

Artificial cerebrospinal fluid (ACSF) was continuously bubbled with a carbogen gas mixture (95% O_2_/5% CO_2_) and maintained at 32°C in a water bath. The slicing solution was pre‐cooled to a slush‐ice state. Five male GRPR‐GFP mice were deeply anesthetized via isoflurane inhalation. To enhance tissue viability, the dorsal skin was clipped and the exposed back musculature was covered with crushed ice prior to rapid decapitation. The lumbosacral spinal cord enlargement was quickly dissected, with extra tissue trimmed from both ends to minimize mechanical damage to the region of interest. The isolated spinal cord was immediately immersed in oxygenated, ice‐cold slicing solution for 1–2 min. The meninges were carefully removed, and the tissue was embedded in a pre‐made, sterilized 3% agar block. The agar block was glued to the vibratome stage, submerged in cold, oxygenated slicing solution, and transverse sections (300 μm thick) were cut using the following parameters: vibration amplitude of 0.80 mm/s and advancing speed of 0.25 mm/s. The entire slicing procedure was performed at approximately 4°C. Each slice was transferred to oxygenated ACSF (32°C) and incubated for recovery for at least 90 min before recording. Continuous gassing was maintained throughout.

#### 2.7.2 Whole‐Cell Patch‐Clamp Recording Procedure

The recording chamber was continuously perfused with oxygenated ACSF at a rate of 2.0–3.0 mL/min using a peristaltic pump, with bath temperature maintained at 32°C ± 0.5°C via a recirculating water bath. Borosilicate glass microelectrodes were pulled and filled with a K^+^‐based intracellular solution. Electrode resistance was confirmed to be between 5–8 MΩ. Under low magnification (×10 objective), the crescent‐shaped SDH region was located. The objective was then switched to high magnification (×40) to visually identify GRPR‐positive neurons based on their green fluorescent protein (GFP) signal. Whole‐cell patch‐clamp recordings were subsequently performed on these targeted neurons.

### 2.8 Role of GABA_A_R Antagonism in the Anti‐Pruritic Effect of Testosterone

Male C57BL/6 mice (6–8 weeks old) were randomly divided into two groups. Both groups received consecutive subcutaneous abdominal injections of testosterone for 3 days. One day prior to behavioral testing, the fur on the nape and back was shaved. Mice were briefly anesthetized with an inhalation anesthesia machine and placed in a prone position. An intrathecal injection (10 μL volume) was administered at the L5‐L6 intervertebral space using an insulin syringe. Mice in Group 1 (Control) received 10 μL of normal saline. Mice in Group 2 (experimental, Bic group) received 10 μL of a bicuculline solution (0.2 μg/μL). Thirty minutes postinjection, after confirming full recovery from anesthesia and normal locomotor activity, an acute itch model was induced via intradermal histamine injection into the nape region. Itch‐related scratching behavior was assessed by counting the number of scratching bouts over a 30‐min observation period.

### 2.9 Statistics and Analysis

All data are presented as mean ± standard error of the mean (Mean ± SEM). Statistical analyses were carried out with GraphPad Prism 9.0. Multiple‐group comparisons were performed via one‐way or two‐way analysis of variance (ANOVA), followed by Tukey's post hoc multiple comparisons test. Unpaired two‐tailed Student's *t*‐test (or Welch's *t*‐test for unequal variances) was used for pairwise comparisons between two groups. Exact *p*‐values together with *F* statistics for two‐way ANOVA are reported in the text and figure legends. Statistical significance was defined as *p* < 0.05.

## 3 Results

### 3.1 Testosterone Inhibits Acute Itch Behavior in Normal Male and Female Mice

Compared to vehicle‐treated control mice, subcutaneous 0.5 mg testosterone markedly suppressed scratching bouts within 30 min post intradermal histamine (His) injection (Figure 1A, *p* < 0.05). The 1.0 mg dose exerted far stronger, highly significant anti‐pruritic inhibition. Although 1.5 mg testosterone also attenuated scratching compared to the control group (Figure 1A, *p* < 0.001), no significant difference was observed between the 0.5 mg and 1.5 mg groups (Figure 1A, *p* > 0.05). Collectively, exogenous subcutaneous testosterone elicited graded anti‐pruritic activity at 0.5–1.0 mg, whereas efficacy declined at 1.5 mg, indicative of a ceiling or partial high‐dose toxic effect instead of a monotonic linear dose–response relationship. The 1.0 mg dose yielded maximal itch suppression and was adopted for all subsequent validation assays.

**FIGURE 1:**
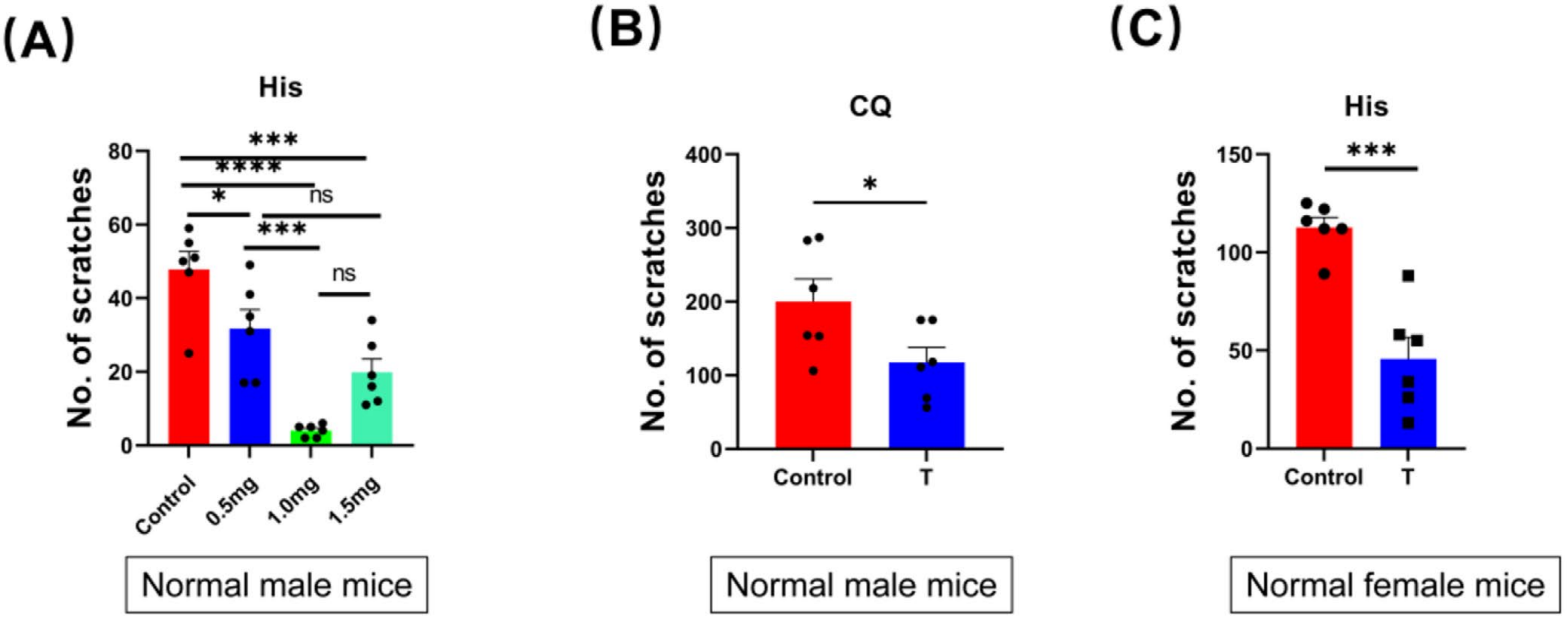
Effect of abdominal subcutaneous injection of testosterone on acute itch. (A) Quantification and comparative analysis of scratching bouts in response to histamine‐induced itch in male mice receiving subcutaneous abdominal injections of testosterone at different concentrations (0.5 mg, 1.0 mg, 1.5 mg) or olive oil (Control group). *n* = 6 per group. *F* = 21.00, *p* < 0.0001, Tukey's post hoc pairwise comparisons. (B) Quantification and comparative analysis of scratching bouts in response to chloroquine‐induced itch in male mice with subcutaneous abdominal injections of 1.0 mg testosterone (T group) or olive oil (Control group). *n* = 6 per group. *F* = 2.19, *p* > 0.05, Unpaired *t*‐test. (C) Quantification and comparative analysis of scratching bouts in response to histamine‐induced itch in female mice administered with subcutaneous abdominal injections of 1.0 mg testosterone (T group) or olive oil (Control group). *n* = 6 per group. *F* = 4.46, *p* < 0.001, Unpaired *t*‐test. ns: No statistically significant difference, *p < 0.05, ***p < 0.001, ****p < 0.0001.

To further characterize this effect across sexes and different pruritogens, we tested the selected testosterone dose (50 μg/μL) in separate models. Mice in the experimental (T) group received subcutaneous abdominal injections of 20 μL testosterone for three consecutive days, while control mice received the vehicle (olive oil). Acute itch was then induced by intradermal injection of either chloroquine (CQ) in male mice or histamine (His) in female mice. The results demonstrated that exogenous testosterone administration effectively inhibited acute itch in both sexes. In male mice, the T group showed a significant decrease in scratching bouts following CQ injection compared to the control group (Figure 1B, *p* < 0.05). Similarly, in female mice, the T group exhibited a markedly reduced scratching response to His injection relative to controls (Figure 1C, *p* < 0.001).

### 3.2 Castrated Male Mice Exhibit Enhanced Itch Sensitivity, Which Is Reversed by Testosterone Supplementation

Seven days after orchiectomy, no statistically significant difference was observed in the number of histamine (His)‐induced scratching bouts between the sham‐operated (ShamV) group and the orchiectomy + olive oil (Orx + olive) group (Figure 2A, *p* > 0.05). In contrast, the orchiectomy + testosterone (Orx + T) group, which received supplementation of 1 mg testosterone per mouse, showed a significant reduction in His‐induced scratching compared to both the ShamV and Orx + olive groups (Figure 2A, *p* < 0.01, *p* < 0.001). These results indicate that itch sensitivity did not change significantly at this early postoperative time point. This unaltered itch sensitivity at postoperative Day 7 likely arises from incomplete depletion of endogenous testosterone, coupled with confounding postsurgical nociception that masks pruritic behavioral readouts.

**FIGURE 2:**
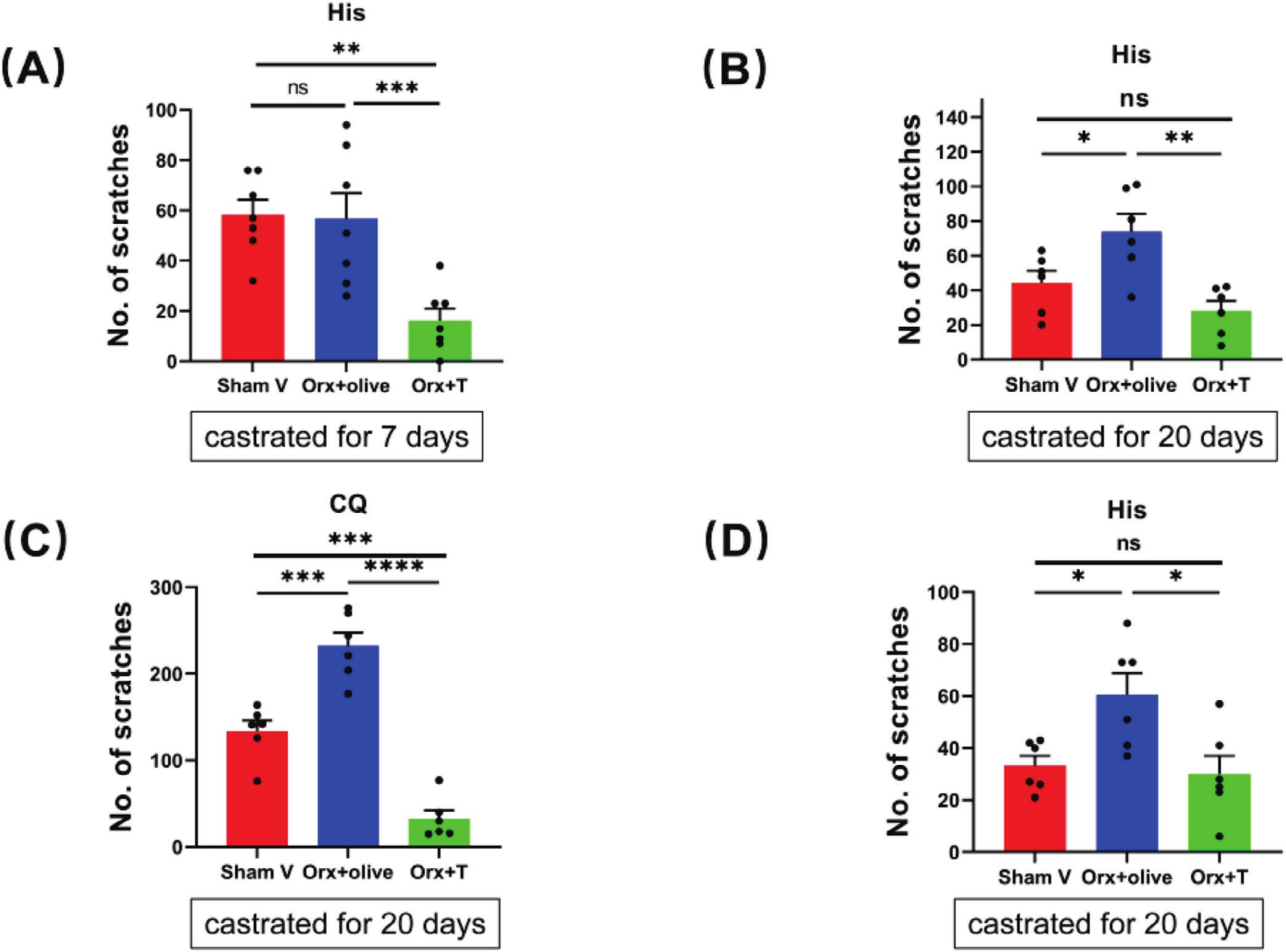
Changes of itch sensitivity in castrated male mice and the effect of testosterone supplementation. (A) Quantification and comparative analysis of histamine (His)‐induced scratching bouts in mice from the sham‐operated group (ShamV), orchiectomized male group (Orx + olive), and orchiectomized male group receiving subcutaneous abdominal injection of testosterone (1 mg per mouse, Orx + T) at 7 days post‐surgery. *n* = 7 per group. *F* = 10.47, *p* < 0.001, Tukey's post hoc pairwise comparisons. (B) Quantification and comparative analysis of His‐induced scratching bouts in mice from the ShamV, Orx + olive, and Orx + T (1 mg) groups at 20 days post‐surgery. *n* = 6 per group. *F* = 8.74, *p* < 0.01, Tukey's post hoc pairwise comparisons. (C) Quantification and comparative analysis of chloroquine (CQ)‐induced scratching bouts in mice from the ShamV, Orx + olive, and Orx + T (1 mg) groups at 20 days post‐surgery. *n* = 6 per group. *F* = 59.41, *p* < 0.0001, Tukey's post hoc pairwise comparisons. (D) Quantification and comparative analysis of His‐induced scratching bouts in mice from the ShamV, Orx + olive, and Orx + T (0.5 mg) groups at 20 days post‐surgery. *n* = *6* per group. *F* = 1.84, *p* < 0.05, Tukey's post hoc pairwise comparisons. ns: no statistically significant difference, *p < 0.05, **p < 0.01, ***p < 0.001, ****p < 0.0001.

By 20 days postorchiectomy, a clear enhancement of itch sensitivity was evident. The number of His‐induced scratching bouts in the Orx + olive group was significantly higher than in the ShamV group (Figure 2B, *p* < 0.05). Supplementation with 1 mg testosterone (Orx + T group) significantly reduced scratching compared to the Orx + olive group (Figure 2B, *p* < 0.01), restoring it to a level not significantly different from the ShamV group (Figure 2B, *p* > 0.05). This suggests that the heightened itch sensitivity following prolonged testosterone depletion can be normalized by exogenous testosterone replacement. A similar but more pronounced pattern was observed with chloroquine (CQ)‐induced itch. At 20 days post‐surgery, the Orx + olive group displayed a highly significant increase in CQ‐induced scratching compared to the ShamV group (Figure 2C, *p* < 0.001). Testosterone supplementation (1 mg) in the Orx + T group markedly reduced this response compared to the Orx + olive group (Figure 2C, *p* < 0.0001). Notably, the scratching frequency in the testosterone‐supplemented group was even significantly lower than that in the intact ShamV group (Figure 2C, *p* < 0.001), indicating a potent suppressive effect of testosterone on CQ‐induced itch that exceeds baseline levels in normal mice.

To investigate a lower dose, orchiectomized mice received 0.5 mg testosterone (Orx + T) for 3 days at the 20‐day time point. Consistent with the higher dose results, the Orx + olive group showed significantly increased His‐induced scratching compared to the ShamV group (Figure 2D, *p* < 0.05). The Orx + T group receiving 0.5 mg testosterone exhibited a significant reduction in scratching compared to the Orx + olive group (Figure 2D, *p* < 0.05), again restoring it to a level indistinguishable from the ShamV group (Figure 2D, *p* > 0.05).

In summary, these results demonstrate that approximately 20 days after orchiectomy, male mice exhibit significantly reduced testosterone levels accompanied by a marked elevation in itch sensitivity to both histamine and chloroquine. Exogenous testosterone supplementation effectively reduces this enhanced itch sensitivity. The increase in sensitivity was more pronounced for chloroquine‐induced itch, and the therapeutic effect of testosterone was correspondingly more significant in this model. These findings confirm a prominent anti‐pruritic role for testosterone, which likely contributes to the observed sexual dimorphism in itch perception.

### 3.3 Intrathecal Administration of the Androgen Receptor Antagonist Flutamide Reverses the Anti‐Pruritic Effect of Testosterone

Compared to the control group (Saline), mice that received an intrathecal injection of testosterone (T) alone showed a significant reduction in the number of histamine (His)‐induced scratching bouts (Figure 3, *p* < 0.001). In contrast, co‐administration of testosterone with its receptor antagonist, flutamide (T + Flu group), markedly increased scratching behavior relative to the T‐only group (Figure 3, *p* < 0.01), effectively abolishing testosterone's suppressive effect on itch. Notably, intrathecal injection of flutamide alone (Flu group) had no significant impact on His‐induced itch compared to the control (Figure 3, *p* > 0.05). These results confirm that the anti‐pruritic action of testosterone is specifically mediated by its binding to androgen receptors (AR).

**FIGURE 3:**
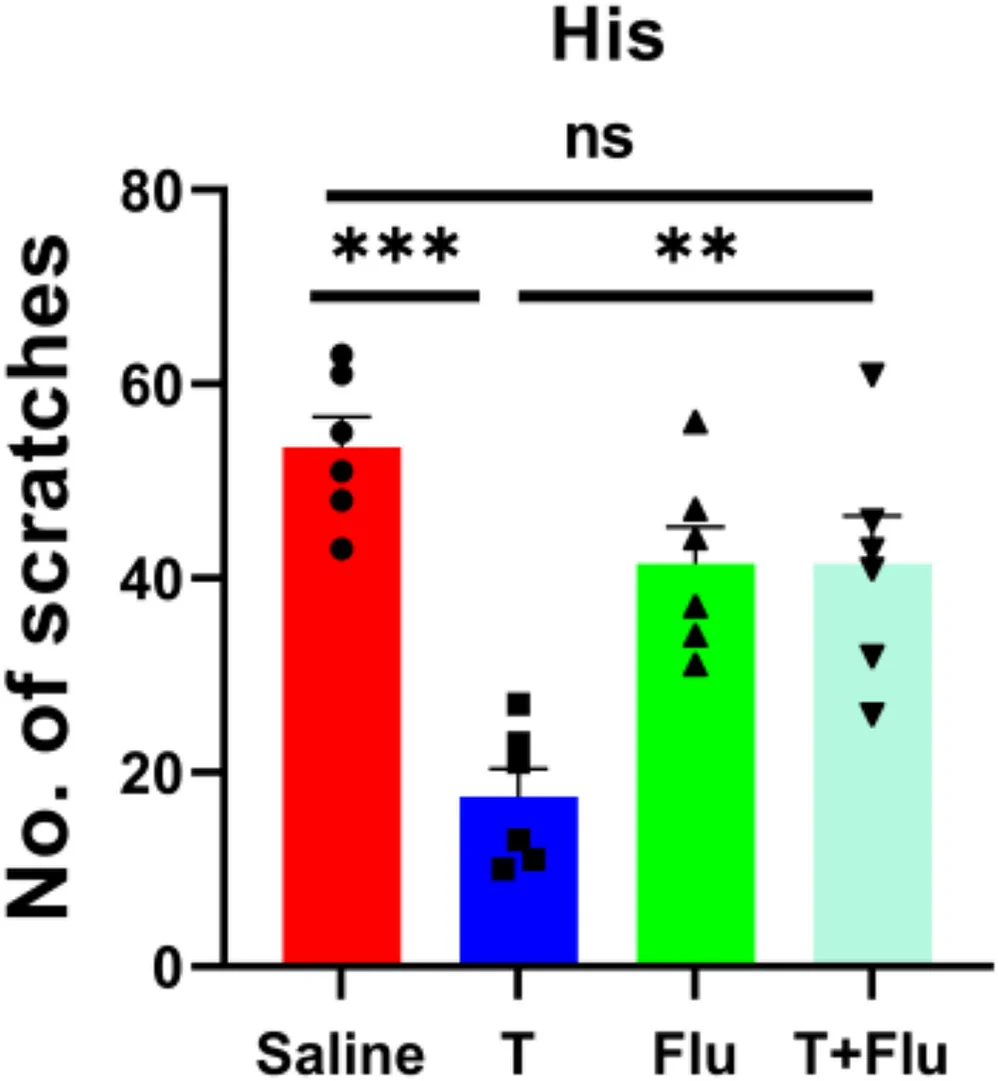
Intrathecal androgen receptor (AR) antagonism abrogates the spinal anti‐pruritic activity of testosterone. Spinal testosterone delivery suppresses histamine‐evoked pruritus via AR‐dependent signaling pathways. Male mice were intrathecally injected with one of four formulations: saline (Control), testosterone only (5 mmol/L, T), the AR antagonist flutamide only (10 mmol/L, Flu), or a mixture of testosterone and flutamide (T + Flu). *n* = 6 per group. *F* = 15.96, *p* < 0.0001, Tukey's post hoc pairwise comparisons. ns: No statistically significant difference, **p < 0.01, ***p < 0.001.

### 3.4 Testosterone Inhibits Chronic Pruritus and Associated Anxiety‐Like Behaviors

Compared with olive oil‐treated DNFB control mice (Figure 4A), subcutaneous testosterone administration significantly reduced spontaneous scratching frequency in both male and female DNFB chronic itch mice, with females showing a more pronounced suppressive effect on scratching behavior (Figure 4B, male: *p* < 0.01; female: *p* < 0.0001). In male mice, total travel distance in the open‐field test (OFT) was significantly elevated (Figure 4C,E, *p* < 0.05), accompanied by a significant increase in the distance traveled within the central zone (Figure 4F, *p* < 0.05). In the elevated plus‐maze (EPM) test, the percentage of time spent in the open arms was significantly increased (Figure 4D,G, *p* < 0.01), although the percentage of entries into the open arms did not differ significantly (Figure 4H, *p* > 0.05). These findings indicate that testosterone not only exerts a pronounced inhibitory effect on chronic itch but also alleviates the anxiety‐like behaviors that accompany it.

**FIGURE 4:**
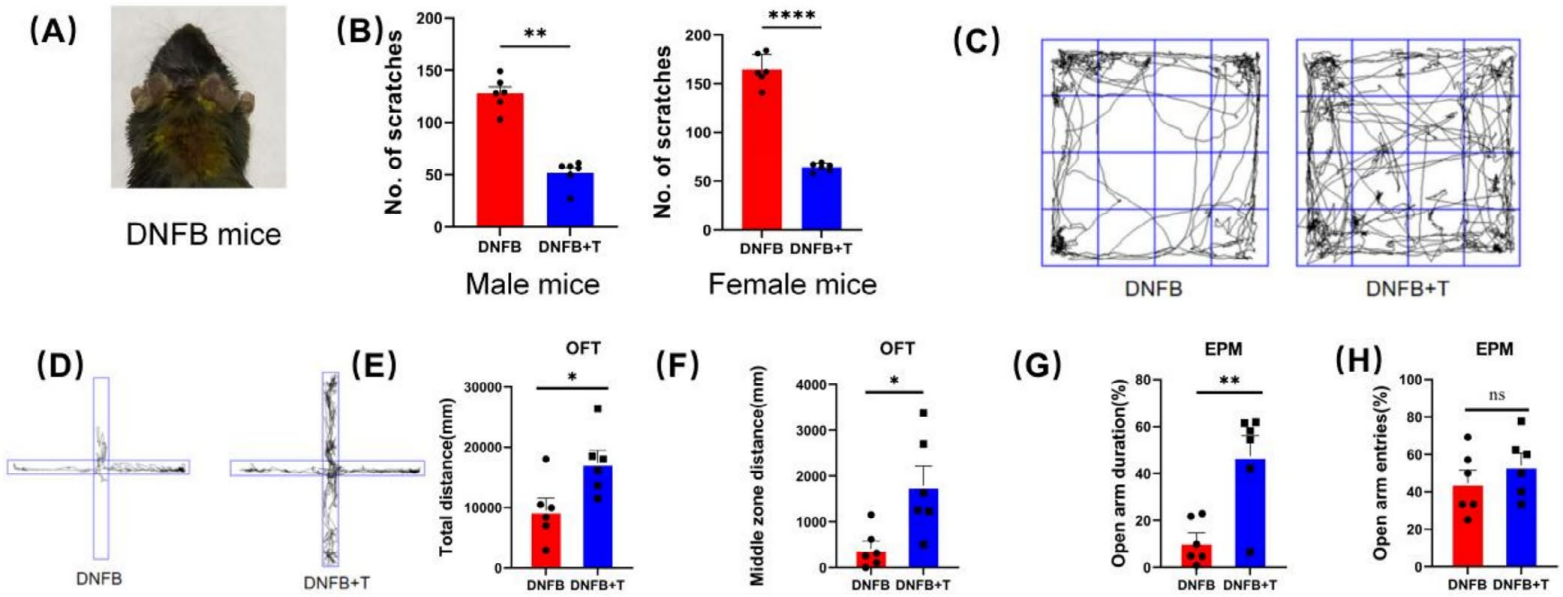
Effects of testosterone on itch and anxiety‐like behaviors in DNFB‐induced chronic itch model. (A) Representative skin lesions in DNFB‐induced chronic itch mice following scratching behavior. (B) Quantification of spontaneous scratching bouts in male and female mice from DNFB and DNFB+T groups. Male: *F* = 1.54, *p* < 0.01, Unpaired *t*‐test, Female: *F* = 15.44, *p* < 0.0001, Welch's *t*‐test. (C‐H) Open‐field test (OFT) and elevated plus‐maze (EPM) behavioral metrics for male mice in the DNFB vehicle and DNFB+T testosterone‐treated groups: (C) OFT movement trajectory traces, (D) EPM movement trajectory traces, (E) total travel distance in the OFT arena, (F) travel distance within the OFT central zone, (G) percentage of time spent in EPM open arms, (H) percentage of entries into EPM open arms. *n* = 6 per group. ns: No statistically significant difference, *p < 0.05, **p < 0.01, ****p < 0.0001. (E) *F* = 1.06, *p* > 0.05; (F) *F* = 6.40, *p* > 0.05; (G) *F* = 5.16, *p* > 0.05; (H) *F* = 1.09, *p* > 0.05. Unpaired *t*‐test.

### 3.5 Androgen Receptors Are Predominantly Expressed on PAX2‐Positive Neurons in the Superficial Dorsal Horn

Double immunofluorescence staining for the androgen receptor (AR) and the inhibitory neuron marker paired box protein 2 (PAX2) yielded the following observations. Sparsely distributed AR‐immunopositive neuronal somata were detected in the mouse SDH (green fluorescence, Figure 5A,D), with a relatively higher density in the superficial laminae (Figure 5A′,A″). In contrast, a large population of PAX2‐immunopositive neuronal somata was observed, particularly concentrated within the superficial laminae (red fluorescence, Figure 5B′,B″). Critically, 72.5% of all AR‐expressing neurons co‐localized with PAX2^+^ inhibitory interneurons (Figure 5D′,D″), indicating that AR is predominantly expressed on inhibitory (likely GABAergic) neurons. DAPI‐stained nuclei exhibited blue fluorescence (Figure 5C′,C″). This finding suggests that testosterone may exert its modulatory effects by acting on GABAergic interneurons via androgen receptors.

**FIGURE 5:**
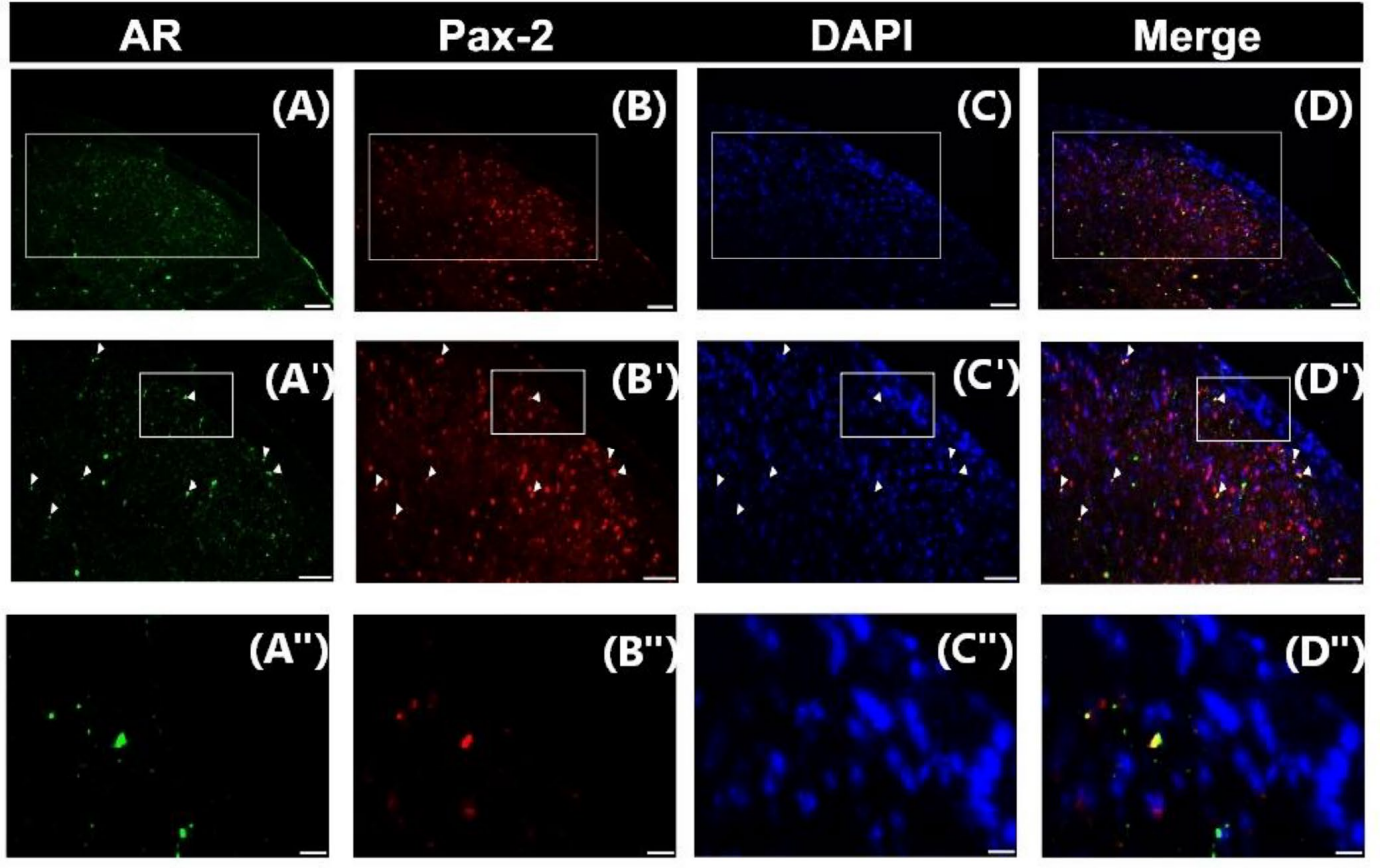
Double staining of AR and PAX2 immunofluorescence. (A) AR‐positive staining. (B) PAX2‐positive staining. (C) DAPI‐positive staining. (D) Merge of A, B, C. (A′, B′, C′, D′) The magnified views of the regions delineated by white boxes in A, B, C, and D, respectively; white arrows point to the AR/PAX2 double‐labeled neurons. (A″, B″, C″, D″) The further magnified views of the regions delineated by white boxes in A′, B′, C′ and D′, respectively. Scale bars = 100 μm (for A–D); 50 μm (for A′–D′);10 μm (forA″–D″).

### 3.6 Testosterone Decreases Excitability of GRPR‐Positive Neurons in the Superficial Dorsal Horn

To electrophysiologically verify our hypothesis that testosterone suppresses itch signaling in gastrin‐releasing peptide receptor‐positive (GRPR^+^) neurons indirectly via activation of spinal inhibitory interneurons, we performed whole‐cell patch‐clamp recordings on GFP‐labeled GRPR^+^ neurons within the superficial dorsal horn of GRPR‐GFP transgenic mice. At a holding potential of −70 mV, bath perfusion with ACSF supplemented with 5 μM testosterone produced no significant changes in the frequency or amplitude of spontaneous excitatory postsynaptic currents (sEPSCs) relative to ACSF‐only control (Figure 6A–C, *p* > 0.05, *n* = 8 neurons). In contrast, when neurons were clamped at 0 mV, testosterone application robustly increased the frequency of spontaneous inhibitory postsynaptic currents (sIPSCs) without altering sIPSC amplitude (Figure 6A,D,E, *p* < 0.05). Notably, co‐perfusion of testosterone with the GABA_A_ receptor antagonist bicuculline fully abolished the testosterone‐evoked elevation in sIPSC frequency (Figure 6A,E, *p* < 0.05).

**FIGURE 6:**
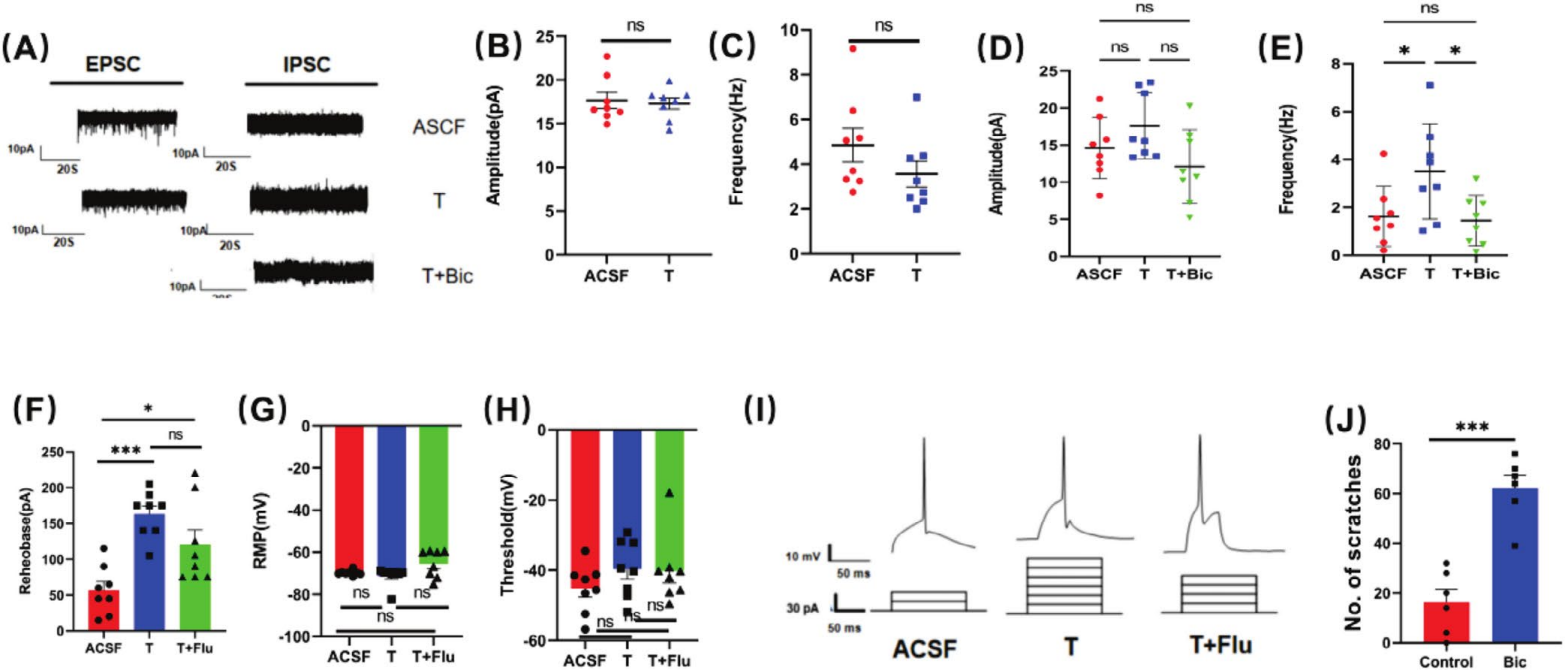
Effect of testosterone on electrophysiological characteristics of GRPR^+^ neurons in spinal dorsal horn. (A) Representative voltage‐clamp recordings of spontaneous excitatory postsynaptic currents (sEPSCs) and spontaneous inhibitory postsynaptic currents (sIPSCs) in GRPR^+^ neurons of the spinal dorsal horn from mice in control (ACSF) group and testosterone‐treated (T) group and bicuculline (Bic) group. (B, C) Statistical graphs showing the changes in sEPSC amplitude (B) and frequency (C) of GRPR^+^ neurons in the ACSF and T groups. (B) *F* = 2.11, *p* > 0.05; (C) *F* = 1.70, *p* > 0.05, Unpaired *t*‐test. (D, E) Statistical graphs showing the changes in sIPSC amplitude (D) and frequency (E) of GRPR^+^ neurons in ACSF, T, and Bic groups. (D) *F* = 1.12, *p* > 0.05, Unpaired *t*‐test; (E) *F* = 12.60, *p* < 0.05, Welch's *t*‐test. (F) Statistical graph of rheobase in GRPR^+^ neurons from mice in ACSF, T, and T + Flu groups. *F* = 4.70, *p* < 0.05, Tukey's post hoc pairwise comparisons. (G) Statistical graph showing the changes in resting membrane potential (RMP) of GRPR^+^ neurons from mice in ACSF, T, and T + Flu groups. *F* = 2.95, *p* > 0.05, one‐way ANOVA. (H) Statistical graph showing the changes in threshold potential of GRPR^+^ neurons from mice in ACSF, T, and T + Flu groups. *F* = 1.05, *p* > 0.05, one‐way ANOVA. (I) Representative evoked action potentials and step currents of GRPR^+^ neurons from mice in ACSF, T, and T + Flu groups. *n* = 8 per group. ns: No statistically significant difference, *p < 0.05, ***p < 0.001. (J) Intrathecal GABA_A_R antagonism reverses testosterone's anti‐pruritic effect. Control group: Intrathecal injection of testosterone plus normal saline; Bic group: Intrathecal injection of testosterone plus bicuculline. *n* = 6 per group. *F* = 1.09, *p* < 0.001, Unpaired *t*‐test. ***p < 0.001.

Under current‐clamp configuration, the rheobase (the minimal current magnitude sufficient to trigger action potentials) of GRPR^+^ neurons was markedly higher in testosterone‐treated slices relative to ACSF controls (Figure 6F,I, *p* < 0.001). Co‐application of the AR antagonist flutamide partially reversed this testosterone‐dependent rheobase elevation (Figure 6F,I, *p* < 0.05 vs. testosterone group).

Resting membrane potential and action potential threshold were comparable across ACSF, testosterone, and testosterone + flutamide groups, with no statistically significant intergroup differences (Figure 6G,H, *p* > 0.05).

Collectively, these results indicate that testosterone does not exert direct regulatory effects on GRPR^+^ neurons. Instead, it suppresses GRPR^+^ neuronal excitability indirectly by enhancing GABAergic inhibitory synaptic input onto these pruriceptive neurons. This testosterone‐triggered elevation in sIPSC frequency was fully abrogated via bath perfusion of bicuculline, confirming that GABA_A_ receptor signaling is required to mediate testosterone's anti‐pruritic activity. In addition, flutamide partially reversed the testosterone‐evoked rise in rheobase and abolished testosterone's suppressive action on GRPR^+^ neuron excitability. Together, these functional findings indirectly confirm that AR and GABAergic machinery co‐localize within the same superficial SDH inhibitory interneurons.

### 3.7 The GABA_A_R Antagonist Bicuculline Reverses the Anti‐Pruritic Effect of Testosterone

The role of GABA_A_R in mediating testosterone's effect was tested pharmacologically. Two groups of male C57BL/6 mice pretreated with subcutaneous testosterone for 3 days received an intrathecal injection of either normal saline (Control group) or the GABA_A_R antagonist bicuculline (Bic group). Acute itch was then induced by intradermal histamine injection. The results showed that the number of histamine‐induced scratching bouts was significantly increased in the Bic group compared to the Control group (Figure 6J, *p* < 0.001). This demonstrates that spinal GABA_A_R blockade fully abolishes testosterone's anti‐pruritic actions. These findings further confirm the proposed mechanism: testosterone likely acts via AR on inhibitory (e.g., GABAergic) neurons to promote GABA release; GABA then acts on GABA_A_R to inhibit GRPR^+^ neurons, thereby suppressing spinal itch transmission.

## 4 Discussion

Mounting clinical and population‐based epidemiological evidence confirms a significantly higher prevalence of pruritus in females (40.7%) than males (38.9%, *p* < 0.001) globally, indicating a fundamental sexual dimorphism in itch perception that may be mediated by sex hormones such as estradiol acting on spinal itch pathways [19, 20]. Although estrogen's pruritic regulatory function has been well characterized, the modulatory effect of the primary androgen testosterone remains largely unelucidated [3, 21]. This study provides comprehensive evidence that testosterone acts as a crucial anti‐pruritic factor and significantly contributes to these observed sex‐based differences.

The key finding of the present research is the anti‐pruritic effects of testosterone. Administration of exogenous testosterone produced a significant suppression of acute itch behaviors in mice, triggered by both histamine and chloroquine. This effect was consistent in both male and female mice, suggesting its potential as a broad therapeutic agent. The anti‐pruritic action of testosterone was specifically mediated through androgen receptor. To verify whether testosterone is a key factor in the sex difference in itch, we conducted castration and testosterone supplementation experiments in male mice. The results showed that the enhanced itch sensitivity became evident only 20 days postsurgery, not at 7 days, indicating a dependence on the gradual depletion of residual testosterone. Crucially, testosterone replacement therapy normalized itch sensitivity in castrated males, confirming its role in maintaining lower itch sensitivity in males. Beyond acute models, testosterone showed promise in mitigating chronic pruritus. In a DNFB‐induced chronic itch model, it reduced spontaneous scratching and improved associated anxiety‐like behaviors. Notably, intermittent testosterone treatment even prevented the development of chronic itch, hinting at prophylactic applications.

Given the complexity of supraspinal pruriceptive circuits and limited available transgenic tools for central neuronal dissection, we focused on the mechanism of testosterone‐induced analgesia on the local circuit of itch transmission and modulation in the SDH, specifically investigating the effects of testosterone on GRPR^+^ neurons, the core component of this local circuit. Electrophysiological recordings revealed that testosterone elevated the frequency of sIPSCs and increased the action potential threshold of GRPR^+^ pruriceptive neurons, consequently reducing their excitability. Testosterone's anti‐pruritic activity relies entirely on GABA_A_R signaling, as this inhibitory effect was fully abrogated both in vivo and ex vivo by the GABA_A_ receptor antagonist bicuculline: intrathecal bicuculline injection completely reversed testosterone's suppression of itch behaviors, and bath perfusion of bicuculline eliminated the testosterone‐triggered rise in sIPSC frequency. These functional results demonstrate that testosterone strengthens GABAergic inhibitory synaptic input onto SDH GRPR^+^ neurons, and further verify that testosterone‐evoked inhibitory currents are mediated exclusively by GABA_A_ receptors rather than glycinergic transmission. Complementary morphological staining confirms that androgen receptor (AR) expression is predominantly localized to PAX2^+^ inhibitory interneurons (including both GABAergic and glycinergic subtypes) within the superficial SDH. Collectively, these lines of evidence support that testosterone acts via AR on PAX2^+^ GABAergic interneurons to enhance GABA_A_‐dependent synaptic inhibition, thereby dampening itch signaling in GRPR^+^ neurons.

Figure 7 summarizes the comprehensive mechanisms elucidated in this study. The sex difference framework demonstrates how estrogen promotes itch while testosterone inhibits it, with testosterone exerting its effects through the spinal circuit mechanism detailed above. Our findings establish a clear link between hormonal regulation and itch perception, highlighting testosterone's role in suppressing itch at both behavioral and cellular levels. Specifically, testosterone activates AR‐positive GABAergic interneurons in the superficial dorsal horn, enhancing GABAergic inhibition onto GRPR^+^ pruriceptive neurons via GABA_A_R. This neural modulation not only explains the hormone's anti‐pruritic effects but also accounts for its ability to reduce anxiety in chronic itch conditions.

**FIGURE 7:**
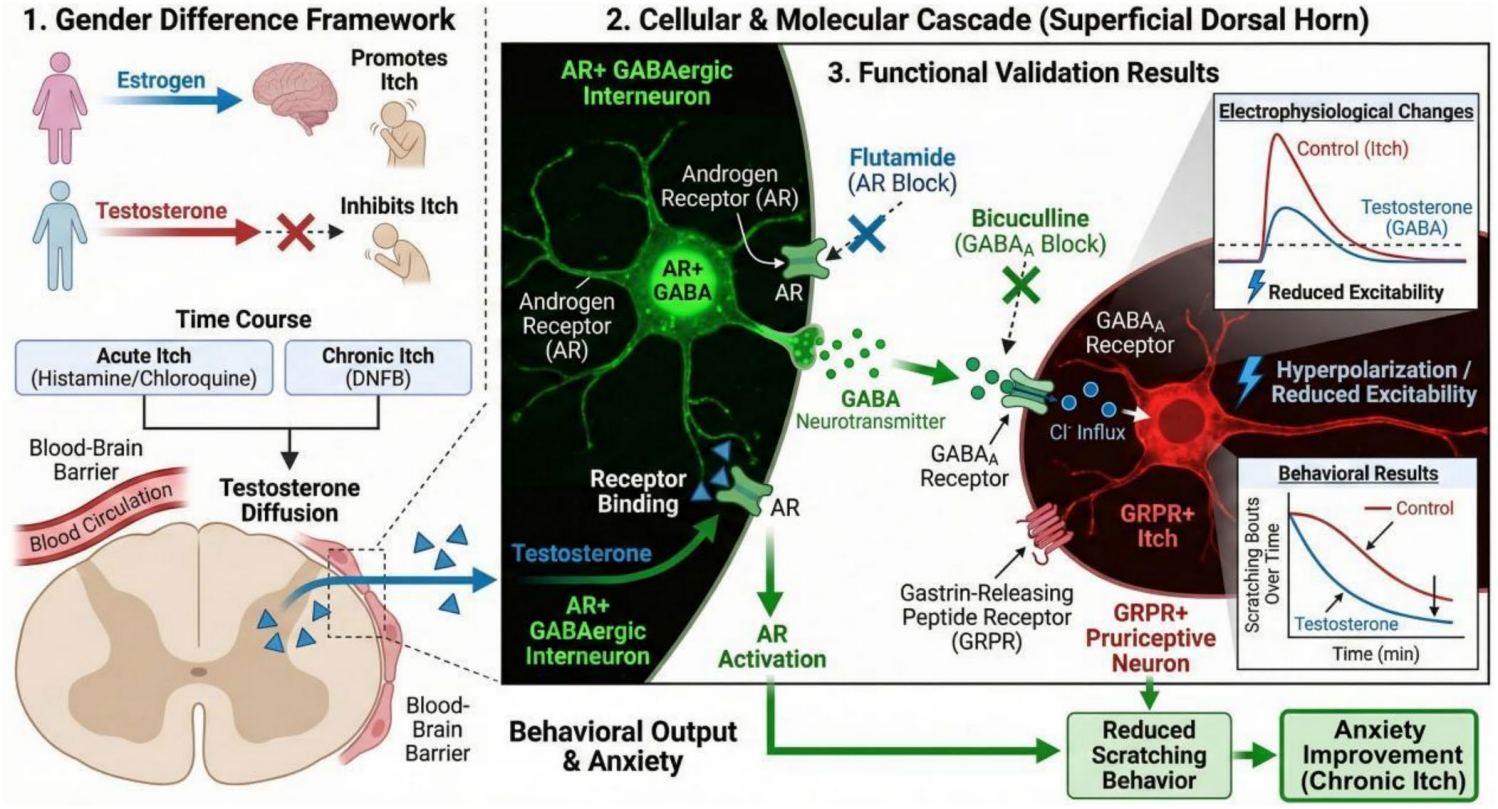
Summary of the comprehensive mechanisms elucidated in this study.

This study establishes testosterone as a critical mediator of sexually dimorphic pruritus in parallel with estrogen, and delineating its spinal neuronal regulatory machinery, this work provides a rigorous framework for developing sex‐specific therapeutic interventions against acute and chronic pruritus.

## Author Contributions

**Luying Chen:** writing – original draft, methodology, investigation, validation. **Xiaodong Wang:** writing – original draft, investigation, data curation, validation, funding acquisition. **Lu Cheng:** investigation. **Pengtao Li:** investigation. **Zhenyu Wu:** writing‐review and editing, supervision, project administration, data curation, funding acquisition, conceptualization. **Hui Li:** writing‐review and editing, supervision, investigation, project administration, funding acquisition, conceptualization.

## Funding

This work was funded by the National Natural Science Foundation of China, (Grant No. 32171137 to H. Li); Natural Science Foundation of Shaanxi Province (Grant No. 2025JC‐YBMS‐183 to Z.‐Y. Wu); Armed Police National Defense Research Project (Grant No. ZZKY2024311 to X.‐D. Wang).

## Ethics Statement

All animal breeding and surgical procedures were approved by the Animal Ethics Committee of the Fourth Military Medical University (Approval No. IACUC‐20210356).

## Conflicts of Interest

The authors declare no conflicts of interest.

## Data Availability

The data that support the findings of this study are available from the corresponding author upon reasonable request.
